# Attentional Modulation of Emotional Conflict Processing with Flanker Tasks

**DOI:** 10.1371/journal.pone.0060548

**Published:** 2013-03-27

**Authors:** Pingyan Zhou, Xun Liu

**Affiliations:** 1 Key Laboratory of Behavioral Science, Institute of Psychology, Chinese Academy of Sciences, Beijing, China; 2 University of Chinese Academy of Sciences, Beijing, China; Cardiff University, United Kingdom

## Abstract

Emotion processing has been shown to acquire priority by biasing allocation of attentional resources. Aversive images or fearful expressions are processed quickly and automatically. Many existing findings suggested that processing of emotional information was pre-attentive, largely immune from attentional control. Other studies argued that attention gated the processing of emotion. To tackle this controversy, the current study examined whether and to what degrees attention modulated processing of emotion using a stimulus-response-compatibility (SRC) paradigm. We conducted two flanker experiments using color scale faces in neutral expressions or gray scale faces in emotional expressions. We found SRC effects for all three dimensions (color, gender, and emotion) and SRC effects were larger when the conflicts were task relevant than when they were task irrelevant, suggesting that conflict processing of emotion was modulated by attention, similar to those of color and face identity (gender). However, task modulation on color SRC effect was significantly greater than that on gender or emotion SRC effect, indicating that processing of salient information was modulated by attention to a lesser degree than processing of non-emotional stimuli. We proposed that emotion processing can be influenced by attentional control, but at the same time salience of emotional information may bias toward bottom-up processing, rendering less top-down modulation than that on non-emotional stimuli.

## Introduction

Researchers have been tackling the relationship between emotion and attention with two questions: how emotion affects attention and how attention modulates emotion. Many studies have addressed the first question, and demonstrated that emotionally salient stimuli can influence attentional processing [1], [2]. For instance, positive faces may influence attention by broadening its focus [3]–[5]. However, only a few studies have addressed the second one and the findings are rather inconsistent.

On one hand, some behavioral studies have indicated that emotion processing is pre-attentive [6]–[11], which takes place relatively quickly, and can be done without conscious awareness [12]. Öhman and colleagues found that fear-relevant search was unaffected by the location of the target in the display and the number of distractors in a visual search task, supporting the notion that emotion processing is pre-attentive and not affected by attentional load [8]. Some imaging studies also provided evidences that emotion processing was independent of attentional resources [13]–[16], implying that processing of emotion is not only automatic but may even be accomplished without consciousness.

On the other hand, growing evidence has demonstrated that emotion processing is modulated by attention [1], [17]–[21]. Gomez-Cuerva and colleagues examined whether sensitivity of emotional expressions was influenced by prior attentional state in a dual-task [22]. Their results showed that sensitivity to negative expressions was significantly lower when the same identity with the negative expression was a distractor (to-be-ignored) in the previous trial than when it was a target (to-be-attended). These results indicated that detection of emotional expressions was affected by prior attentional states. Other imaging studies have indicated that emotion processing is modulated by attentional control [23]–[29]. For example, Pessoa and colleagues found that the amygdala was significantly activated when emotional faces were attended and such activation disappeared when participants diverted attention from emotional faces to lines [24]. However, Vuilleumier and colleagues found that activation of amygdala was not influenced by attention [14]. Thus the question of whether emotion processing is modulated by attention is still the subject of extensive debate and warrants further investigation.

Many studies demonstrated that emotion processing was influenced by attention have mostly focused on spatial orientation of attention, for example the dot-probe task [30], visual search task [8], [31] and dual-task paradigm [22]. However, fewer studies have examined whether emotion processing was affected by attention in executive control. Emotional Stroop task was frequently used to investigate emotional conflict in previous studies [32]–[37]. Egner and colleagues aimed to investigate whether the neural circuitry recruited for emotional conflict resolution was the same as that of non-emotional conflict [32]. Faces were presented with either positive or negative expression superimposed word “HAPPY” or “FEAR” to create emotionally congruent and incongruent stimuli. Participants were required to identify the facial expressions. Behavioral results revealed that reaction time (RT) of emotionally incongruent condition was significantly longer than that of emotionally congruent condition [35]. Although these findings indicated that emotion processing can be modulated by attention, little is known about how this effect is compared to attentional modulation of the non-emotional information. Thus, the current study examined attentional modulation of conflicts rising from emotional and non-emotional stimuli, and compared these effects directly to elucidate whether and to what degree emotion processing is influenced by attention.

The current study adopted a classic stimulus response compatibility (SRC) task, the Eriksen flanker task [38], to investigate whether emotion was modulated by attention in conflict monitoring and resolution of emotional stimuli [12], [39]–[41]. We designed two experiments using male and female faces with neutral, happy and fearful expressions as stimuli. In the first experiment, the neutral faces were artificially painted in red and blue, therefore the flanker faces may be congruent or incongruent from the target face in either color or gender or both dimensions. The aim of combining two dimensions was to generate two levels of processing, task relevant and task irrelevant, so that top-down attentional modulation of stimuli could be examined. When participants were required to identify the color of the target, color processing was task relevant and gender processing was task irrelevant. Vice versa, when the task was to identify the gender of the target, gender processing became task relevant and color processing was task irrelevant. We could examine whether the color or gender processing was modulated by task-directed top-down control. If so, by varying color or gender as the task dimension, color conflict in the relevant task would result in a larger SRC effect than in the irrelevant task, and so would gender conflict. In the second experiment, we replaced the color dimension with the emotion dimension, using gray scale faces with either happy or fearful expression. And there were two types of emotional conflicts, a happy target flanked by fearful faces and a fear target flanked by happy faces. When participants were required to identify the emotion of the target, emotion processing was task relevant and gender processing was task irrelevant. Vice versa, when the task was to identify the gender of the target, gender processing was task relevant and emotion processing was task irrelevant. We investigated whether emotion or gender was also modulated by top-down attention feedback. If emotion was processed without attention, consumed little or no attentional capacity [42], then the SRC effects for the relevant and irrelevant tasks would not be significantly different. Alternatively, we would observe a larger SRC effect in the relevant task than in the irrelevant task for emotional conflict. On the other hand, salience may render emotional stimuli less susceptible to attention modulation than other non-emotional stimuli, thus resulting in smaller SRC effects of emotional stimuli. To investigate how these factors influenced emotion processing, we also contrasted the SRC effects between the relevant and irrelevant tasks in two experiments.

## Materials and Methods

### Participants

Twenty-four healthy college students (11 female; aged 19–27) volunteered to participate in this study. All of them gave informed written consent, which was approved by the Ethical Committee of Institute of Psychology, Chinese Academy of Sciences. All of them were right-handed and had normal or corrected-to-normal vision. Each student was paid to compensate for the time and travel cost.

### Stimuli and tasks

Participants performed two sets of experiments, which adopted the flanker task. The stimuli were 12 faces (6 women), generated in FaceGen Modeller 3.4. Three different expressions of each face were created, neutral, happy, and fearful. To avoid using teeth to detect expression, both happy and fearful faces were with the mouth open. Colored faces were converted to gray scale in Photoshop and faces were cropped to an oval shape. Then all faces were adjusted to have equal average skin luminance. Next, gray scale faces were artificially painted in red and blue colors. The gray scale faces were rated by additional 24 participants prior to the study, using a 5-point scale, with 1 as most negative and 5 as most positive in one rating and with 1 as most masculine and 5 as most feminine in another rating. One-way ANOVA found that emotional valence ratings were significantly different across three expressions [*F*(1, 23) = 403.48, *p*<0.01]. Post hoc comparisons indicated that ratings for all three expressions differed from each other. Paired t-test revealed that gender ratings were also significantly different for male and female faces [*t* (1, 23)  = 16.92, *p*<0.01].

**Figure 1:**
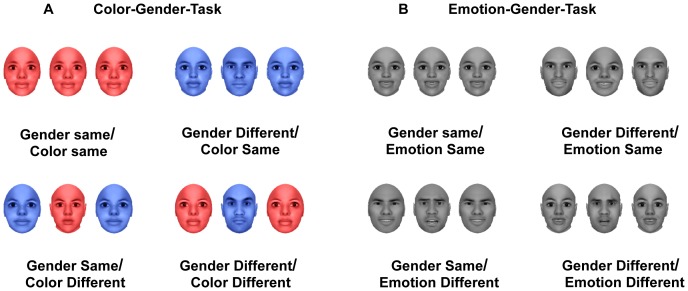
Experimental designs and stimuli for Experiments 1 and 2. A. Color-Gender-Task. B. Emotion-Gender-Task.

In Experiment 1, the stimuli were a row of three colored faces with neutral expression (Figure 1A). Participant’s task was to identify either the color or the gender of the central face ignoring the surrounding distractors. The identity of the flanker and target were randomly selected from a pool of 12 faces (6 men and 6 women), and the selection of each identity as the target or flanker was counterbalanced. The color task and gender task were counterbalanced across blocks within the experiment. Based on the relationship between the target and flankers across the color and gender dimensions, there were four different stimulus conditions: color different / gender different, color different / gender same, color same / gender different, color same / gender same. Prior to the task, participants were trained to identify the task relevant dimension (color or gender) of the face by pressing the left (F) or right (J) key. Key mapping were counterbalanced across participants. Stimuli that may lead to conflicting responses from two dimensions were excluded. For example, if the color of a face led to a right key response but the gender led to a left key response, it was removed from the list. Post-study debriefing suggested that no participant was aware of this combination of the target properties.

During the task, the central face (target) was flanked by two faces (distractors). Each face subtended a visual angle of approximately 2.80°×3.65° in width and height at a viewing distance of 60 cm. The distance between the center of the target face and the center of each flanker face was 3.08°. To prevent participants from adopting strategies to visually filter out the flanker, the flankers and target were randomly shifted horizontally at the same time within a visual angle of about 5.61°. All stimuli were displayed on a dark background. Following a central fixation of 100 ms, target and flankers were displayed for 1400 ms simultaneously. Each trial ended with a blank screen of 500 ms. Participants were instructed to respond as quickly and accurately as possible. The task session contained 4 blocks, each including 128 trials. There were equal numbers of compatible trials and incompatible trials, resulting in 64 trials for each condition in one block. At the beginning of each block, participants were instructed whether to perform the color or gender judgment, the order of which was counterbalanced within participants.

The procedure of Experiment 2 was similar to that in Experiment 1, except that the color dimension was replaced by the emotion dimension (Figure 1B). Stimuli were a row of three gray scale faces with emotional expressions. Neutral faces were not used. Therefore there were also four types of stimuli: emotion different/gender different, emotion different/gender same, emotion same/gender different, emotion same/gender same.

## Results

The RT and accuracy were summarized below for the Color-Gender-Task (Table 1) and Emotion-Gender-Task (Table 2), respectively. RTs beyond ±3 standard deviations (SDs) were removed from the mean in all conditions, 3.71% for Experiment 1 and 5.22% for Experiment 2. For all analysis of variance (ANOVA) tests, the significance level was set at p<0.05. Error bars represent 95% confidence intervals, which were computed by means of a normalization procedure [43], [44].

**Table 1:** Reaction time and Accuracy of the Color-Gender-Task.

Task	Color Task		Gender Task	
Stimulus	RT (ms)	Accuracy (%)	RT (ms)	Accuracy (%)
GDCD	548.65	97	608.02	97
GDCS	521.22	98	613.73	98
GSCD	544.56	97	592.63	99
GSCS	510.3	98	595.03	99

GDCD - color different / gender different; GSCD - color different / gender same; GDCS - color same / gender different; GSCS - color same / gender same.

**Table 2:** Reaction time and Accuracy of the Emotion-Gender-Task.

Task	Emotion Task		Gender Task	
Stimulus	RT (ms)	Accuracy (%)	RT (ms)	Accuracy (%)
GDED	674.27	95	646.14	97
GDES	658.63	96	645.84	97
GSED	670.94	95	634.59	98
GSES	657.36	96	626.1	98

GDED - emotion different / gender different; GSED - emotion different / gender same; GDES - emotion same / gender different; GSES - emotion same / gender same.

### Color-Gender-Task

**Figure 2:**
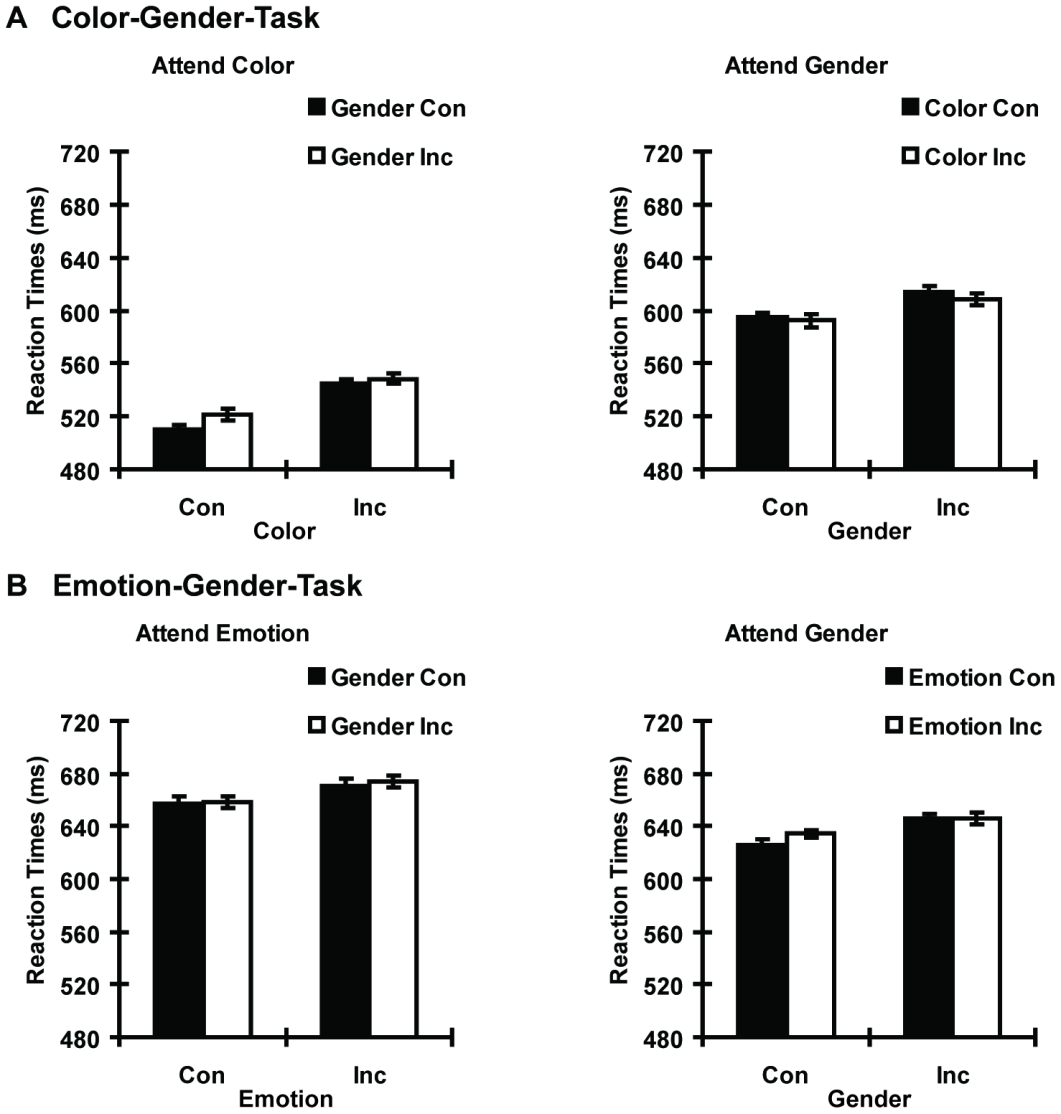
Reaction time as a function of stimuli and tasks for Experiments 1 and 2. A. Color-Gender-Task. B. Emotion-Gender-Task.

A 2 (task: attend color or gender) × 2 (color congruency) × 2 (gender congruency) repeated measure ANOVA was applied to RTs. The results revealed significant main effects in task [*F*(1, 23) = 85.50, η_p_^2^ = 0.79, *p*<0.01], color congruency [*F*(1, 23) = 48.55, η_p_^2^ = 0.68, *p*<0.01], and gender congruency [*F*(1, 23) = 31.78, η_p_^2^ = 0.58, *p*<0.01]. RT of attending to the gender was significantly longer than RT of attending to the color. Incongruency between the target and flankers in either color or gender slowed the performance. Both interactions of color congruency and task [*F*(1, 23) = 52.64, η_p_^2^ = 0.70, *p*<0.01] and of gender congruency and task [*F*(1, 23) = 14.35, η_p_^2^ = 0.38, *p*<0.01] were significant. Post hoc comparisons indicated that color SRC effect was significant when participants attended to the color (task relevant, 30.85 ms difference between color incongruent trials and color congruent trials) and disappeared when participants attended to the gender (task irrelevant, –4.06 ms difference). Gender SRC effects were significant when the gender was either task-relevant or task-irrelevant. However, the former effect (task relevant, 17.05 ms difference between gender incongruent trials and gender congruent trials) was greater than the latter (task irrelevant, 7.51 ms difference). All other interactions were non-significant (Figure 2A).

**Figure 3:**
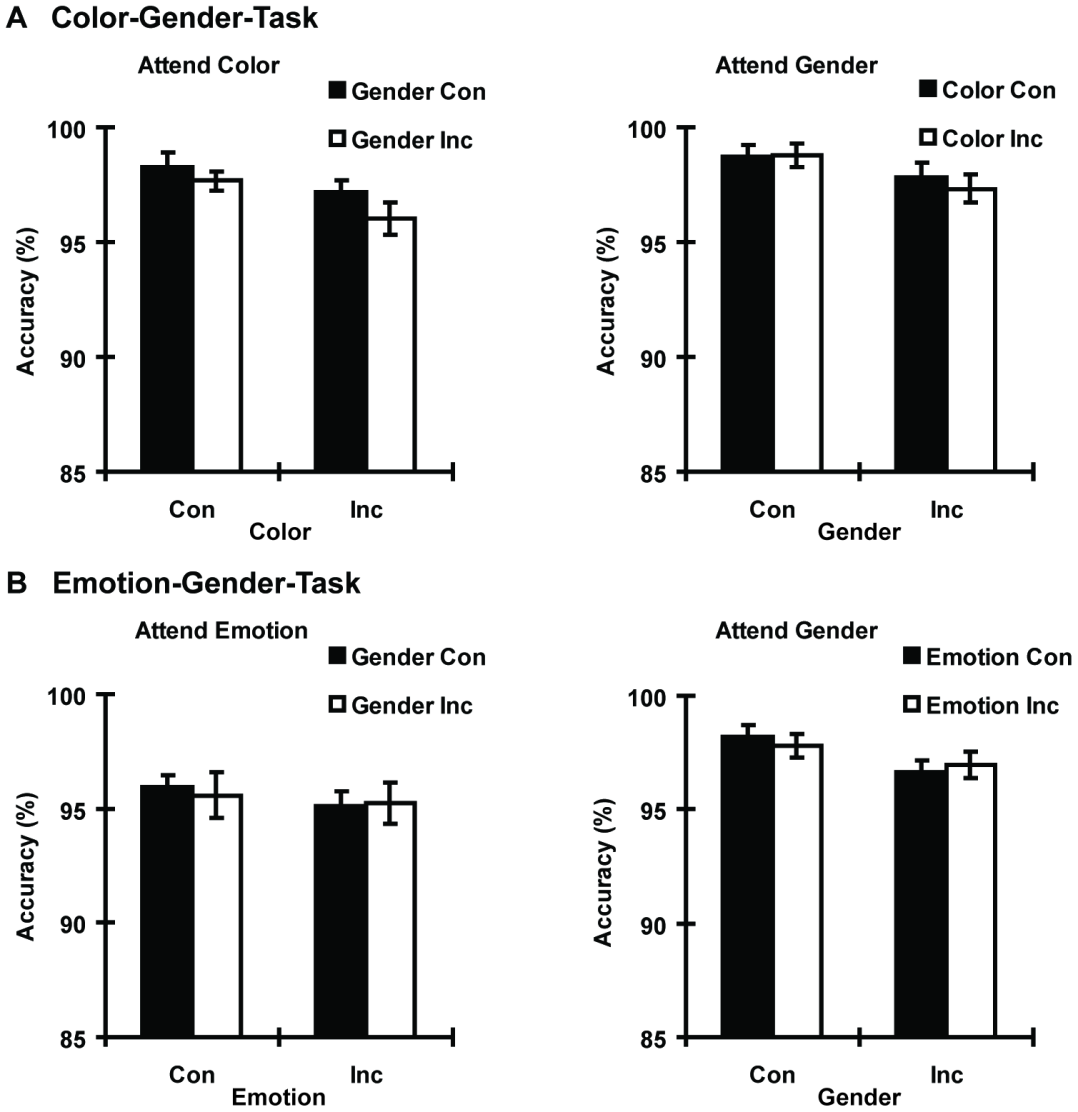
Accuracy as a function of stimuli and tasks for Experiments 1 and 2. A. Color-Gender-Task. B. Emotion-Gender-Task.

For accuracy, main effects of both task [*F*(1, 23) = 11.64, η_p_^2^ = 0.34, *p*<0.01] and gender congruency [*F*(1, 23) = 13.51, η_p_^2^ = 0.37, *p*<0.01] were significant. The main effect of color congruency was marginally significant [*F*(1, 23) = 3.89, η_p_^2^ = 0.15, *p* = 0.06]. Accuracy of attending to the gender was significantly higher than accuracy of attending to the color. The interaction between color congruency and task was significant [*F*(1, 23) = 4.70, η_p_^2^ = 0.17, *p*<0.05]. Post hoc comparisons indicated that color SRC effect was significant when participants attended to the color (task relevant, 1.36% difference), but was not significant when participants attended to the gender (task irrelevant, 0.19% difference). All other interactions were non-significant (Figure 3A).

### Emotion-Gender-Task

A 2 (task: attend emotion or gender) × 2 (emotion congruency) × 2 (gender congruency) was applied to the RTs. The results revealed significant main effects in task [*F*(1, 23) = 7.23, η_p_^2^ = 0.24, *p*<0.05], emotion congruency [*F*(1, 23) = 16.07, η_p_^2^ = 0.41, *p*<0.01], and gender congruency [*F*(1, 23) = 16.22, η_p_^2^ = 0.41, *p*<0.01]. RT of attending to the gender was significant shorter than RT of attending to the emotion. Incongruency in either emotion or gender increased RTs significantly. Both interactions of emotion congruency and task [*F*(1, 23) = 7.07, η_p_^2^ = 0.24, *p*<0.05] and of gender congruency and task [*F*(1, 23) = 12.58, η_p_^2^ = 0.35, *p*<0.01] were significant. Post hoc comparisons indicated that emotion congruency effect was significant when participants attended to the emotion and disappeared when participants attended to the gender. Gender congruency effect was significant when participants attended to the gender, but not when participants attended to the emotion. Both emotion and gender SRC effects were larger in the relevant task than in the irrelevant task. Emotion incongruent trials caused an RT increase of 14.61 ms than emotion congruent trials for the relevant task and 4.4 ms increase for the irrelevant task. Gender processing, similar to emotion processing, was modulated by task-directed attentional control, with 15.64 ms and 2.3 ms of SRC effects for the relevant and irrelevant tasks, respectively. All other interactions were non-significant (Figure 2B).

For accuracy, there was a main effect for task [*F*(1, 23) = 4.55, η_p_^2^ = 0.17, *p*<0.05] with a higher accuracy of attending to the emotion than attending to the gender. The main effect of emotion congruency was not significant [*F*(1, 23) = .87, η_p_^2^ = 0.04, n.s.]. The main effect of gender congruency was marginally significant [*F*(1, 23) = 3.58, η_p_^2^ = 0.14, p = 0.07]. The interaction of emotion congruency and task was not significant [*F*(1, 23) = 0.64, η_p_^2^ = 0.03, n.s.]. The interaction of gender congruency and task was significant [*F*(1, 23) = 4.28, η_p_^2^ = 0.16, p<0.05]. Post hoc comparisons indicated that gender congruency effect was significant when participants attended to the gender, but was not significant when participants attended to the emotion. All other interactions were non-significance (Figure 3B).

### Comparison of SRC effects as a function of stimuli and tasks

**Figure 4:**
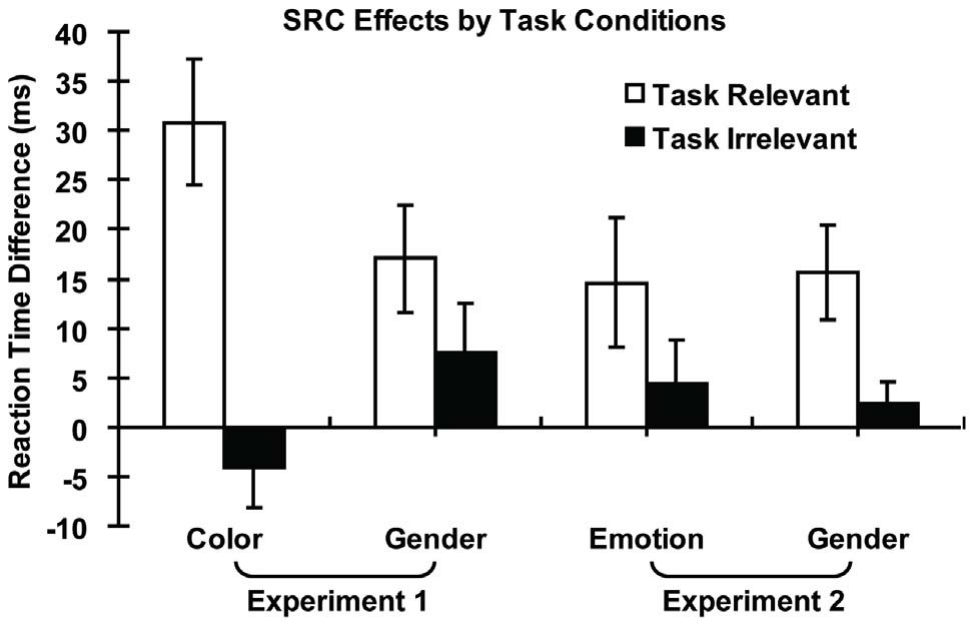
SRC effects (RT difference between incongruent and congruent conditions) as a function of stimuli and tasks across both experiments.

In order to investigate whether attentional modulation of emotion was different from that of gender and color, we conducted a 2 (task: relevant, irrelevant) × 4 (stimuli: color, emotion, gender in two experiments) repeated-measure ANOVA on SRC effects, which were calculated as the differences in RTs between congruent and incongruent conditions (see Figure 4). The results revealed significant main effect of task [*F*(1, 23) = 71.63, η_p_^2^ = 0.76, *p*<0.01] and interaction between task and stimulus [*F*(1, 23) = 10.31, η_p_^2^ = 0.31, *p*<0.01]. The main effect of stimulus was not significant [*F*(1, 23) = 1.05, η_p_^2^ = 0.04, *p*>0.05] Post hoc comparisons showed that the difference between task-relevant and task-irrelevant SCR effects on RTs (i.e. task modulation of the SRC effect) was significantly larger for color than for gender or emotion. Task modulation of emotion SRC effect was not significantly different from that of gender SRC effect. This implied that processing of the gender and emotion was modulated by attention to a lesser degree relative to color processing.

## Discussion

In the present study, we addressed questions of whether emotion processing is modulated by attention and whether attentional modulation of emotional stimulus processing differs from that of non-emotional stimulus processing. The current data revealed that emotion congruency effect was significant when it was task relevant and disappeared when it was task irrelevant, indicating that emotion processing requires some degree of attentional control.

On one hand, our findings showed that emotion processing was modulated by attention. Previous studies found that attentional resource was limited so that selective attention to one stimulus impacts on processing of the other stimulus presented simultaneously [24], [40]. One possible explanation is the biased competition model of attention proposed by Desimone and Duncan [45]. According to the model, the competition is biased towards the stimulus in two ways, bottom-up salience capture and top-down attentional regulation. Stimulus processing is facilitated when it is attended. Even physically non-salient stimulus can win the competition and receive preferential processing with limited resources [45]–[48]. Pessoa et al. argued that emotion processing was enhanced when emotional stimuli was attended and acquired priority in competition, similar to the processing of non-emotional stimuli. Processing of emotion was attenuated or eliminated when emotion became task irrelevant, suggesting that emotion processing was modulated by top-down attention regulation [40], [49].

Using flanker tasks with facial expressions as stimuli, attentional modulation on emotion processing was evaluated. We also included non-emotional conflict processing (e.g., color and face gender) to serve as the baseline for comparison. Bindemann et al. found that only one face can be processed within capacity limit for target-distractor tasks [50]. However, the current study found that when three faces were presented at the same time, SRC effects were observed indicating simultaneous processing of all faces. This discrepancy may be due to the reason that the distance between the target and flankers was much smaller in the current study (0.28°) than that in Bindemann et al.’s study (1.2°). Eriksen et al. also found that the distance between the target and flankers was critical to the SRC effect, with larger distance resulting in smaller effect [38]. Previous studies found that sensitivity for discriminating color was higher when participants selectively attended to color than when they divided attention among color and other properties, indicating that color processing was modulated by attention [51]–[54]. Egner et al. found that fusiform face area (FFA) was significantly activated when face processing was task relevant and no activation of FFA when faces became task irrelevant [14], [55]–[57]. The findings suggested that face processing was also modulated by attention. We also found that color and gender conflict processing were modulated by top-down attentional control in the Color-Gender-Task, similar to previous evidences [52]. In addition, we found that congruency effects for both color and gender attenuated when the conflicting information became task irrelevant, further supporting that processing of these perceptual properties was under the influence of top-down attentional modulation. In the Emotion-Gender-Task, emotional congruency effect showed similar patterns: it was significant when emotion was task relevant and disappeared when emotion became task irrelevant. These findings seemed to weigh in that emotion processing was under the influence of attentional modulation.

On the other hand, our data also showed that processing of emotion or gender was affected by attention to a lesser degree than color processing. Some researchers suggested that salient stimuli could win competition for neural resources through bottom-up mechanism. For example, salient stimuli gain prioritized processing because of their privileged access to attentional resources [45], [49], [58]. Abundant evidences suggest that emotional information receives preferential processing because it can easily capture attention [31], [59]–[64]. Previous studies found that although RT of locating emotional expressions increased as the number of distractors increased in a visual search task, the increasing rates were smaller for the negative and positive emotions than for the neutral expression [65], [66].

Many studies also indicated that faces could receive preferential processing in comparison to other non-biological stimuli [67]–[69]. Vuilleumier et al. investigated how attention affected processing of emotion in patients who had right parietal damages, causing them to neglect the left spatial space [70]. Faces in the left visual field were missed less often than oval shapes and patients extinguished positive and negative expressions much less than neutral expressions. These findings suggest that emotional stimuli captured attention more easily than faces and objects [71]. Therefore processing of emotional expressions and faces (e.g., gender identity) was influenced by attention to a lesser degree in comparison to processing of neutral stimuli, because their salience helps to gain priority in bottom-up processing. From an evolutionary perspective, this is beneficial to survival and social adaptation for humans.

One limitation of the current study was that the viewing distance was not rigorously maintained by participants throughout the experiment, even though they were instructed to maintain the head-to-screen distance. This could slightly change the separation of target and distractors in visual angle if participants leaned forwards or backwards during the session. However, unless participants strategically and systematically changed the viewing distance for the congruent and incongruent conditions (i.e. the changes were not random), this slight variation in visual angle and separation between target and distractors would not have affected SRC effects drastically. Another limitation was that we did not apply any algorithm to manipulate the spatial frequency spectrum of the stimuli, although all stimuli used were created using FaceGen Modeller with a set of consistent parameters. We did not intend to examine the spatial frequency effect on emotional expression processing. Nonetheless, conflicts of different dimensions (i.e. color, gender and emotion) might be confounded with their differences in spatial frequency, which could have affected the SRC effects.

In conclusion, the present study demonstrated that conflict processing of emotional information was modulated by attention, although to a lesser degree as compared to that of non-emotional stimuli. These findings suggest that emotion processing can be influenced by top-down attentional control, but at the same time the salience of emotional information may bias toward bottom-up processing, rendering less top-down modulation than that on non-emotional stimuli.
